# HCG-Activated Human Peripheral Blood Mononuclear Cells (PBMC) Promote Trophoblast Cell Invasion

**DOI:** 10.1371/journal.pone.0125589

**Published:** 2015-06-18

**Authors:** Nan Yu, Wenjie Yan, Tailang Yin, Yaqin Wang, Yue Guo, Danni Zhou, Mei Xu, Jinli Ding, Jing Yang

**Affiliations:** 1 Reproductive Medical Center, Renmin Hospital of Wuhan University, Wuhan, People’s Republic of China; 2 Department of Obstetrics and Gynecology, Tongji Hospital, Tongji Medical College, Huazhong University of Science and Technology, Wuhan, People’s Republic of China; Medical Faculty, Otto-von-Guericke University Magdeburg, Medical Faculty, GERMANY

## Abstract

Successful embryo implantation and placentation depend on appropriate trophoblast invasion into the maternal endometrial stroma. Human chorionic gonadotropin (hCG) is one of the earliest embryo-derived secreted signals in the peripheral blood mononuclear cells (PBMC) that abundantly expresses hCG receptors. The aims of this study were to estimate the effect of human embryo–secreted hCG on PBMC function and investigate the role and underlying mechanisms of activated PBMC in trophoblast invasion. Blood samples were collected from women undergoing benign gynecological surgery during the mid-secretory phase. PBMC were isolated and stimulated with or without hCG for 0 or 24 h. Interleukin-1β (IL-1β) and leukemia inhibitory factor (LIF) expressions in PBMC were detected by enzyme-linked immunosorbent assay and real-time polymerase chain reaction (PCR). The JAR cell line served as a model for trophoblast cells and was divided into four groups: control, hCG only, PBMC only, and PBMC with hCG. JAR cell invasive and proliferative abilities were detected by trans-well and CCK8 assays and matrix metalloproteinase (MMP)-2 (MMP-2), MMP-9, vascular endothelial growth factor (VEGF), tissue inhibitor of metalloproteinase (TIMP)-1, and TIMP-2 expressions in JAR cells were detected by western blotting and real-time PCR analysis. We found that hCG can remarkably promote IL-1β and LIF promotion in PBMC after 24-h culture. PBMC activated by hCG significantly increased the number of invasive JAR cells in an invasion assay without affecting proliferation, and hCG-activated PBMC significantly increased MMP-2, MMP-9, and VEGF and decreased TIMP-1 and TIMP-2 expressions in JAR cells in a dose-dependent manner. This study demonstrated that hCG stimulates cytokine secretion in human PBMC and could stimulate trophoblast invasion.

## Introduction

Successful embryo implantation and placentation depend on the appropriate invasion of fetus-derived trophoblasts into the maternal endometrial stroma [1–3], a process that is initiated during the mid-secretory phase of the menstrual cycle. An insufficient trophoblastic invasion capacity causes embryo implantation dysfunction, early abortion, preeclampsia, and other complications [4].

At the embryonic implantation site, the human embryo buries within the maternal endometrium by 12 days after ovulation and becomes surrounded by maternal blood, which contains peripheral blood mononuclear cells (PBMC) [5]. During decidualization, uterine immune cells appear to dramatically increase and account for at least 15% of all cells in the decidua from the early pregnancy through term [6]. Maternal PBMC interact directly with the trophoblasts and then return to the systemic circulation. To date, several cytokines and chemokines have been identified as being expressed in and even secreted by fetal cytotrophoblasts and decidual stroma, and the corresponding receptors are expressed in the maternal immune cells [7, 8]. Therefore, it was speculated that both hCG and immune cells contribute to the cross-talk between mother and embryo [6].

Circulating mononuclear cells derived from women in early pregnancy were shown to enhance trophectoderm invasion of the murine embryo [9]. These PBMC were also demonstrated to promote BeWo cell invasion by secreting soluble factors [10]. More importantly, when PBMC derived from non-pregnant women were incubated with hCG, the production of chemoattractive factors increased to promote murine embryo and BeWo cell invasion [9]. These findings suggest that hCG alters PBMC functions to facilitate embryo invasion. Moreover, Ideta et al. [11] reported that the administration of autologous PBMC into the uterine horn improves pregnancy rates after bovine embryo transfer. From these findings, we proposed the new hypothesis that peripheral immune cells receive signals from the conceptus in the early stage of pregnancy and activate these PBMC at the implantation site that then secrete cytokines and chemokines to regulate trophoblast invasion and endometrial differentiation to support embryonic implantation at the maternal–fetal interface.

To examine this hypothesis, we investigated the effects of PBMC derived from non-pregnant mice that were co-cultured with hCG for 24 h on embryo implantation and pregnancy rate. As a result, the intrauterine administration of mouse PBMC activated by hCG reinforced the expression of endometrial leukemia inhibitory factor (LIF) and vascular endothelial growth factor (VEGF) in mice with embryonic implantation dysfunction, suggesting that hCG-induced PBMC activation promotes embryonic implantation by regulating endometrial receptivity [12]. In this study, we focus on the physiological role of the interaction between maternal PBMC and trophoblasts at the early implantation site, which is not yet thoroughly understood. Hence, the objectives of this study are to: (i) examine the impact of hCG stimulation, one of the most important embryonal signals, on PBMC function derived from non-pregnant women; and (ii) investigate the effect of these activated PBMC on the invasion capacity of human choriocarcinoma JAR cells line and the underling mechanisms.

## Materials and Methods

### Cells and culture conditions

JAR cells, a continuous cell line established from a human choriocarcinoma, were obtained from the American Type Culture Collection and maintained in Roswell Park Memorial Institute 1640 medium (RPMI 1640) supplemented with 10% fetal bovine serum (FBS), penicillin 100 IU/mL, and streptomycin 100 μg/mL. The cells were maintained as monolayers in 25 cm^2^ flasks at 37°C under 5% CO_2_ in air with high humidity.

### Preparation of PBMC

Human PBMC were prepared as described previously [13]. Volunteers were recruited among healthy non-pregnant women (secretory phase, cycle day 18–24, n = 30) with a regular menstrual cycle. Human PBMC were isolated from 10 mL of venous blood using a Ficoll-Paque PLUS centrifuge as described previously [14]. After the centrifugation, PBMC were collected from the interphase layer and washed four times with RPMI 1640 medium. PBMC (1 × 10^7^ cells/mL) suspended in RPMI 1640 supplemented with 10% (v/v) FBS were incubated with or without hCG (10 IU/mL) for 0 or 24 h in tissue culture dishes at 37°C under 5% CO_2_ in air with high humidity. We strictly obeyed the Declaration of Helsinki for Medical Research involving Human Subjects during the project and obtained written consent from all subjects. This study was approved by the Ethics Committee of Renmin Hospital, Wuhan University (No. WHR 324571).

### Cell invasion assay

Invasion was performed on Matrigel–coated trans-well inserts (6.5 mm; Costar) containing polycarbonate filters with 8-mm pores as described previously [15]. Briefly, the inserts were pre-coated with 50 μL of Matrigel matrix 1 mg/mL at 37°C for 4 h for gelling. JAR cells (1 × 10^5^ in 200 μL of serum-free media) were plated in the upper chamber, whereas the lower well were filled with culture medium (with 10% FBS), culture medium with hCG (10 IU/mL), culture medium with PBMC (1 × 10^7^ cells/mL), and culture medium with PBMC and hCG, respectively. After 24-h incubation, the cells on the Matrigel side of the insert were removed by a cotton swab. The inserts were then fixed in 4% paraformaldehyde for 10 min at room temperature and stained with hematoxylin. Cells invaded to the other side of the insert were counted under a light microscope (Olympus BX51) in five random fields at a magnification of 400×. The assay was repeated three times, and the results were represented as means of invasion percentage (%) ± standard error of the mean in the cell invasion compared with the control.

### Cell proliferation assay

A Cell Counting Kit 8 (CCK8) cell proliferation assay was used to evaluate cell proliferation according to the manufacturer’s instructions. JAR cells were seeded in 96-well plates at a density of 5 × 10^4^/well and grown at 37°C for 24 h to be attached. The cells were subsequently placed in culture medium, culture medium with hCG (10 IU/mL), culture medium with PBMC (1 × 10^7^ cells/mL), and culture medium with PBMC and hCG for 24, 48, or 72 h. After 10 μL of WST-8 dye ([2-(2-methoxy-4-nitrophenyl)-3- (4-ni-trophenyl))-5- (2,4-disulfophenyl)-2H- tetrazolium, monosodium salt was added to each well, the cells were incubated at 37°C for 2 h, and the absorbance at 450 nm was finally determined (Victor3 1420 Multilabel Counter).

### Enzyme-linked immunosorbent assay

The concentrations of interleukin-1β (IL-1β) and LIF in PBMC-conditioned medium were determined using a commercial human enzyme-linked immunosorbent assay kit based on appropriate and validated sets of monoclonal antibodies. The assays were performed as instructed by the manufacturer. The supernatants of the conditioned medium from PBMC (5 × 10^6^ cells/mL) with or without hCH (10 IU/mL) were collected at 0 and 24 h. Cytokine levels were related to those of the control incubation without the supernatant of the conditioned medium. All experiments were performed in triplicate and measured at 450 nm (Victor3 1420 Multilabel Counter).

### Western blotting

Proteins were extracted from JAR cells cultured in: culture medium (control), culture medium with hCG 10 IU/mL; 400 μL, 800 μL, and 1600 μL of the culture supernatants of PBMC with hCG for 24 h; and whole cell lysis buffer (50 mM HEPES, 150 mM NaCl, 1 mM EGTA, 10 mM sodium pyrophosphate, 1.5 mM MgCl_2_, 100 mM NaF, 10% glycerol, and 1% Triton X-100) containing an inhibitor cocktail (1 mM phenylmethylsulfonylfluoride, aprotinin10 mg/mL, and 1 mM sodium orthovanadate). Culture media were concentrated using a Heraeus Fresco 21 centrifugal filter. The protein concentration was measured using the Bradford method of protein quantification by spectrophotometry at 595 nm. Thirty micrograms of denatured proteins per well was subjected to sodium dodecyl sulfate–polyacrylamide gel electrophoresis according to standard protocols. Separated proteins were transferred electrophoretically onto a polyvinylidene fluoride blotting membrane. After being blocked with 5% skim milk for 1 h at room temperature, the membrane was sequentially incubated with primary antibodies against matrix metalloproteinase (MMP)-2 (1:1000; ab3715), MMP-9 (1:1000; ab38898), VEGF (1:1000; sc-507) tissue inhibitor of metalloproteinase (TIMP)-1 (1:1000; ab77847), TIMP-2 (1:1000; ab53730), and β-actin (1:1000; 1854) overnight at 4°C and washed three times for 10 min per wash with Tris-buffered saline with Tween-20 (TBST). A subsequent incubation with monoclonal horseradish peroxidase–conjugated antibody was performed for 1 h at room temperature in 5% skim milk, and three times of washes with TBST were performed. Immunoreactive bands were detected using enhanced chemiluminescence.

### Reverse transcription and real-time polymerase chain reaction

Total RNA was isolated from cultured PBMC and JAR cells using Trizol extraction reagent, from which single-strand cDNA was synthesized using RevertAid First Strand cDNA Synthesis kit. The purity of the extracted RNA was photometrically tested, and the ratio of the optical density (OD) at 260/280 nm was 1.8–2.0. A total of 20 μL of the reaction contained 1 μg of template RNA (2 μL), 1 μL of oligo (dT)_18_ primer, 9 μL of diethylpyrocarbonate-treated water, 4 μL of 5× reaction buffer, 1 μL of RiboLock RNase inhibitor (20 U/μL), 2 μL of 10 mM dNTP Mix, 1 μL of RevertAid M-MuLV Reverse Transcriptase (200 U/μL). The reactions were incubated at 65°C for 5 min, 42°C for 60 min, and 70°C for 5 min and then instantly cooled on ice.

Specific primers were used for the polymerase chain reaction (PCR). The primers were designed with computer assistance according to GenBank. The sequence of IL-1β was (up stream: 5’- ATG GCT TAT TAC AGT GGC -3’ and down stream: 5’- GTA GTG GTG GTC GGA GA -3’, 221bp); LIF was (up stream: 5’- AGT GCC AAT GCC CTC TTT AT -3’ and down stream: 5’- CCA AGG TAC ACG ACT ATG CG -3’, 167bp); MMP-2 was (up stream: 5’- CGG CCG CAG TGA CGG AAA -3’ and down stream: 5’- CAT CCT GGG ACA GAC GGA AG -3’, 232bp); MMP-9 was (up stream: 5’- GAC GCA GAC ATC GTC ATC CAG TTT -3’ and down stream: 5’- GCC GCG CCA TCT GCG TTT-3’, 207bp); VEGF-A was (up stream: 5’- CTC TAC CTT CCA CCA TGC CAA G -3’ and down stream: 5’- AGA CAG GCT ATC TGG GAC CGC AGG GAC TGC CAT CCA TGA ACT TCA CCA CTT C -3’, 241bp); TIMP-1 was (up stream: 5’- TAC TTC CAC CGG TCC CAC AAC C -3’ and down stream: 5’- GGC TAT CTG GGA CCG CAG GGA CTG CCA -3’, 151bp); TIMP-2 was (up stream: 5’- AAA CGA CAT TTA TGG CAA CCC TAT C -3’ and down stream: 5’- ACA GGA GCC GTC ACT TCT CTT GAT G -3’, 205bp); β-actin was (up stream: 5’-TCC TTC TGC ATC CTG TCA GCA-3’ and down stream: 5’-CAG GAG ATG GCC ACT GCC GCA -3’, 300bp). PCR was performed using a 7500 Real Time PCR System in a final volume of 20 μL containing 10 μL of 2× SYBR Premix Ex Tap, 2 μL of 2.5 mM primer, 0.4 μL of 50× ROX Reference Dye, 1 μL of DNA template, 6.6 μL of dH_2_O. The following cycling conditions were used: 95°C for 30 sec, 40 cycles at 95°C for 5 sec, and 60°C for 34 sec, 95°C for 15 sec, 60°C for 60 sec, and 95°C for 15 sec.

### Statistical analysis

All experiments were performed at least in triplicate. The data were statistically analyzed and expressed as mean ± standard error of the mean. Means were analyzed by one-way analysis of variance followed by the Newman-Keuls test. Significance was accepted at values of P < 0.05. The statistical analyses were performed using SPSS ver. 13.0 for Windows.

## Results

### HCG-induced expression of IL-1β and LIF in cultured PBMC

**Fig 1:**
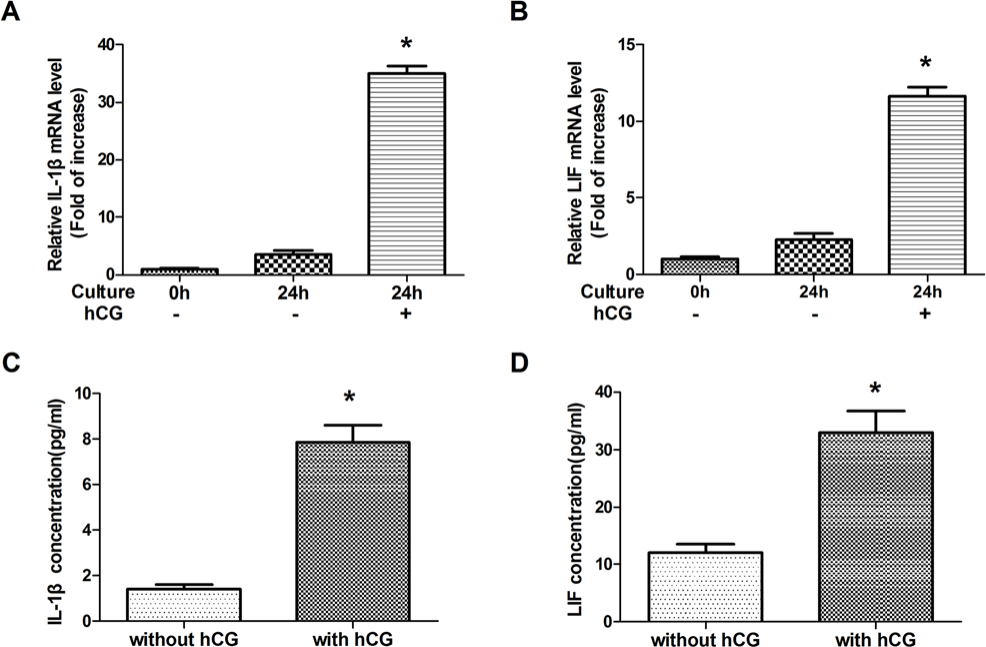
The effect of human chorionic gonadotropin (hCG) on the gene expression of interleukin (IL)-1β and leukemia inhibitory factor (LIF) in peripheral blood mononuclear cells (PBMC) and the concentration of these cytokines in the medium of PBMC after 24-h culture. Changes in the relative amounts of IL-1β (A) and LIF (B) mRNA in human PBMC cultured for 0 h (non-cultured) or 24 h. Data are expressed as mean ± standard error of the mean (SEM) of relative IL-1β or LIF mRNA levels (fold of increase). *, P < 0.05 compared with the other two groups. IL-1β (C) and LIF (D) secretion levels from PBMC were measured at 24 h after culturing with or without hCG. Data are presented as mean ± SEM of triplicate experiments.*, P < 0.05.

To examine the effect of hCG on IL-1β and LIF mRNA expression and protein secretion, PBMC were cultured with or without hCG for 0 or 24 h. A real-time PCR assay showed that IL-1β and LIF transcripts were expressed in human cultured PBMC (Fig 1A and 1B). The expressions of IL-1β mRNA (P < 0.05) and LIF mRNA (P < 0.05) were significantly increased in human PBMC cultured with hCG for 24 h compared to those of PBMC cultured without hCG for 0 and 24 h. The supernatant IL-1β and LIF levels from PBMC cultured with hCG for 24 h were higher than that from PBMC cultured without hCG for 24 h (Fig 1C and 1D). These results showed that hCG significantly increased IL-1β and LIF expression levels in human PBMC.

### Effect of hCG-activated PBMC on trophoblast invasion

**Fig 2:**
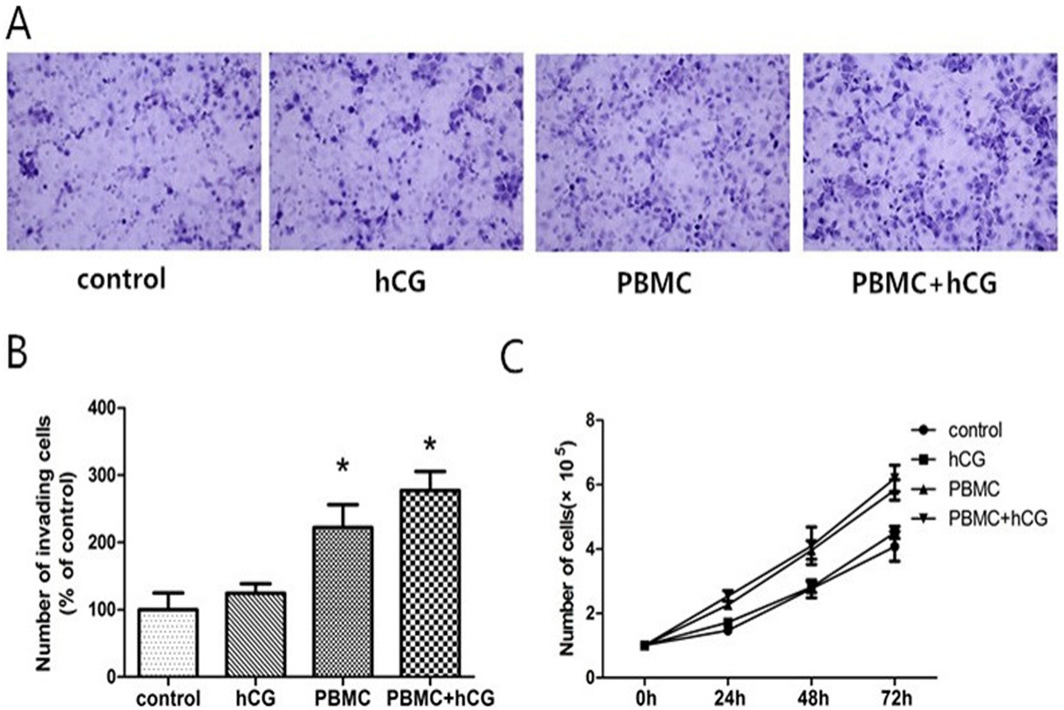
The effect of peripheral blood mononuclear cells (PBMC) activated by human chorionic gonadotropin (hCG) on JAR cell invasion and proliferation. (A) Invasion assays were of JAR cells resuspended in culture medium (control), 10 IU/mL hCG, PBMC, and PBMC with hCG, respectively. Representative microphotographs showing fixed and stained JAR cells on the bottom side of an invasion membrane from invasion assay. Magnification, ×400. (B) The invasion assay data are expressed as the mean fold change ± standard error of the mean of the number of invasive cells in each condition relative to the respective control. *P < 0.05. (C) Proliferation assay of JAR cells in the four groups over 3 culture days (P > 0.05).

To explore the possible roles of hCG-activated PBMC in human trophoblast invasion, Matrigel-coated trans-well inserts were used. In the cell invasion assay, PBMC in the culture media of the lower chambers of the invasion well increased JAR cell invasion index by 2.3-fold at 24h compared to controls (P < 0.05). PBMC with hCG was found to increase invasion most significantly, by 2.8-fold (P < 0.05) (Fig 2A and 2B). In contrast, hCG only had no significant effects on invasive capacity (P > 0.05). These findings suggest that PBMC activated by hCG can enhance the invasion activity of trophoblast cells.

### Effect of hCG-activated PBMC on trophoblast proliferation

To analyze whether hCG-activated PBMC influences JAR cell proliferation, cumulative cell numbers were determined in culture medium, hCG, PBMC, and PBMC with hCG for 24, 48, or 72 h (Fig 2C). The PBMC and PBMC with hCG groups had little effect on JAR cell proliferation with a small tendency toward promoting proliferation that did not reach statistical significance (P > 0.05). There were no statistically significant changes in proliferation at 24, 48, or 72 h of incubation among these four group. These findings suggest that PBMC activated by hCG have no effect on JAR cell proliferation.

### Effect of activated PBMC on MMP-2, MMP-9, VEGF, TIMP-1, and TIMP-2 expressions in trophoblast cells

**Fig 3:**
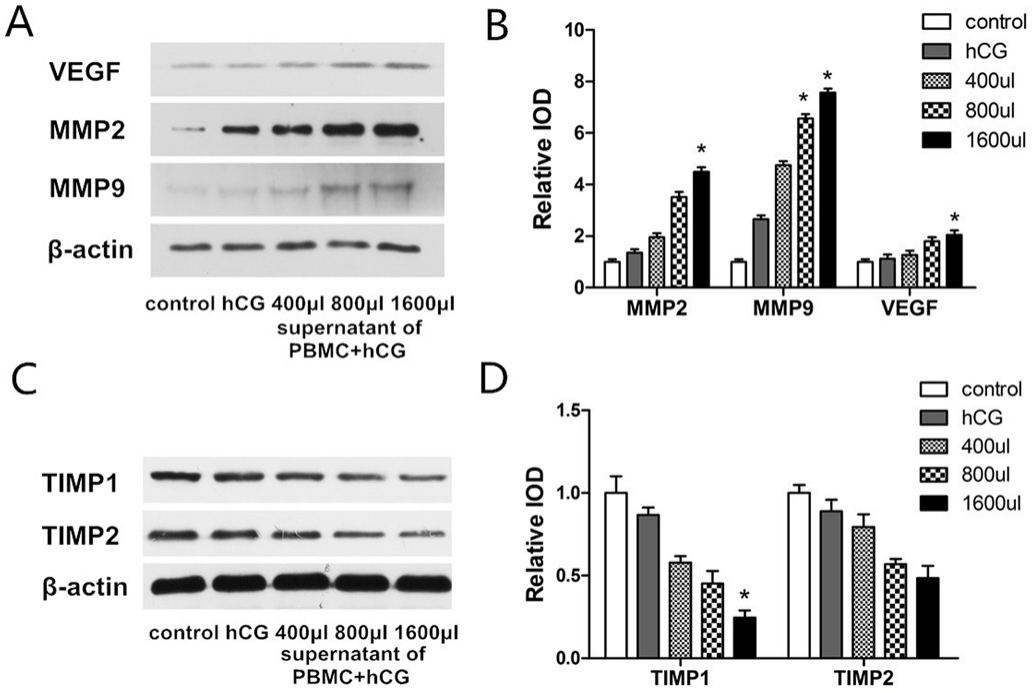
Effect of peripheral blood mononuclear cells (PBMC) activated by human chorionic gonadotropin (hCG) on the protein expression of matrix metalloproteinase (MMP)-2, MMP-9, vascular endothelial growth factor (VEGF), and tissue inhibitor of metalloproteinase (TIMP)1 and TIMP-2 in JAR cells by western blotting assays. (A) Protein expression of MMP-2, MMP-9, VEGF, TIMP-1, and TIMP-2 in control JAR cells or those cultured in hCG, 400, 800, or 1600 μL culture medium supernatant of PBMC with hCG. (B) The intensity of protein expressions of JAR cells compared to control cells using western blot analysis. Data are expressed as mean ± standard error of the mean of triplicate experiments. *, P < 0.05 compared with the control and hCG groups.

To elucidate the potential effector molecules involved in PBMC activated by hCG-induced cell invasion, MMP-2, MMP-9, VEGF, TIMP-1, and TIMP-2 expressions were investigated using western blotting and real time-PCR (Fig 3). The protein expressions of MMP-2, MMP-9, and VEGF were significantly increased in the JAR cells treated with 800 or 1600 μL of culture medium supernatant of PBMC with hCG compared to that in control cells and those treated with hCG (P < 0.05). The expression increased in a concentration-dependent manner (Fig 3A and 3B). TIMP-1 and TIMP-2 protein expression levels were significantly decreased in the JAR cells treated with 1600 μL of culture medium supernatant of PBMC with hCG compared to those in the control and hCG groups (P < 0.05) (Fig 3C and 3D).

**Fig 4:**
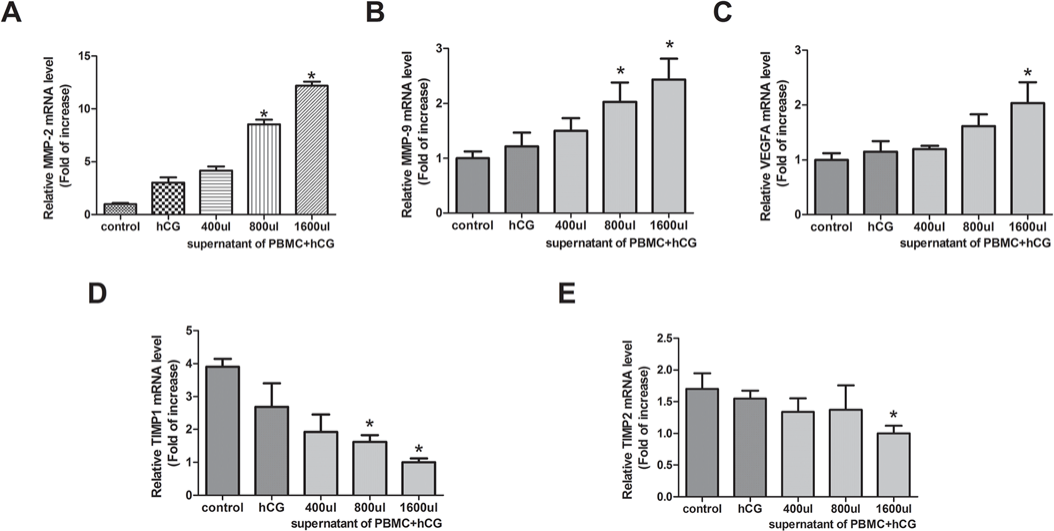
The effect of peripheral blood mononuclear cells (PBMC) activated by human chorionic gonadotropin (hCG) on mRNA expression of matrix metalloproteinase (MMP)-2, MMP-9, vascular endothelial growth factor (VEGF), and tissue inhibitor of metalloproteinase (TIMP)-1 and TIMP-2 in JAR cells by real-time polymerase chain reaction. The mRNA expression of MMP-2, MMP-9, VEGF, TIMP-1, and TIMP-2 in control JAR cells and those cultured in hCG, 400, 800, or 1600 μL of culture medium supernatant of PBMC with hCG. Data are expressed as mean ± standard error of the mean of triplicate experiments. *, P < 0.05 compared with the control and hCG groups.

Real-time PCR analysis showed that MMP-2, MMP-9, VEGF, TIMP-1, and TIMP-2 mRNA were expressed in JAR cells (Fig 4). Compared with the control and hCG-treated groups, MMP-2, MMP-9, and VEGF mRNA expression levels in 800 or 1600 μL of the culture medium supernatant of PBMC with hCG group normalized by β-actin expression levels were significantly increased (P < 0.05), while TIMP-1 and TIMP-2 mRNA expression levels in the PBMC with hCG group were significantly decreased compared with those in the control and hCG-treated groups (P < 0.05). These results suggest that PBMC activated by hCG may regulate trophoblast invasion by upregulating MMP-2, MMP-9, and VEGF expressions and downregulating TIMP-1 and TIMP-2 expressions.

## Discussion

After the embryo attaches to the endometrial epithelial cells, it invades the endometrial stroma by degrading the maternal surrounding tissue and destroying the endometrial vessels. During this process, the circulatory system of maternal blood around the embryo becomes established [9]. Although the interaction between PBMC in the maternal blood and the invading embryo is an inevitable event in the early stage of embryo implantation, its physiological significance is not yet fully understood. In this study, we demonstrated that hCG as a major embryonal signal stimulates the secretion of cytokines in human PBMC, which could enhance the ability of trophoblast cells to invade the extracellular matrix *in vitro*, which is accompanied by increased MMP-2, MMP-9, and VEGF expressions and decreased TIMP-1 and TIMP-2 expressions. This finding suggests that the initial interaction of the invading embryo with PBMC at the implantation site is involved in the regulation of early embryo invasion.

The glycoprotein hormone hCG is one of the most important signaling molecules produced by peri-implantation human embryos and trophoblast cells [16]. At the implantation site, hCG production by human embryos begins at an early stage of development and is secreted into the maternal blood. This early appearance of hCG suggests that it may exert paracrine effects regulating embryo implantation and early placental development [17]. In fact, hCG/luteinizing hormone receptors are found in virtually all maternal tissues in close proximity to the embryo, including the endometrium and maternal immune cells [7, 8].

Lin et al. [18] reported that T-lymphocytes from pregnant women expressed hCG/luteinizing hormone receptor genes and that hCG has immunoregulatory properties that alter human lymphocytes functions [8, 19]. Previous studies on the effects of PBMC on murine embryos invasion showed that the promoting effect of PBMC derived from women in early pregnancy was significantly greater than that of PBMC obtained from non-pregnant women, indicating that there are some functional differences in PBMC between pregnant and non-pregnant women [10]. Hiroshi [14] also found that co-culture of PBMC and luteal cells derived from pregnant women increased the production of Th-2–related cytokines IL-4 and IL-10, which contribute to embryo–mother cross-talk via systemic circulation, induce endometrial differentiation, and promote embryo implantation. Moreover, Nakayama showed that hCG could enhance the promoting effect of PBMC on murine embryo invasion *in vitro* [9]. Thus, the biological activity of PBMC is considered to be changed by hCG, a major embryonic signal, to promote embryo implantation.

To investigate the signaling pathway from the embryo to PBMC, we examined the effect of hCG on cytokine expression by PBMC. Our study showed that LIF and IL-1β expressions are significantly increased in human PBMC incubated with rhCG for 24 h compared with that in PBMC incubated without rhCG, a finding that was similar to those in previous studies in mice and bovine [12, 20]. In addition, Ideta et al. [11] cultured bovine PBMC with hCG for 24 h and confirmed elevated production of cytokines such as IL-1α, IL-1β, and IL-8. Several cytokines, LIF, IL-1, IL-8, IL-11, reportedly promote embryonic invasion in the uterus [2, 21–23]. This leads us to the concept that hCG, which is an important embryonal signal, can activate these PBMC *in vitro* and enhance the secretion of cytokines and chemokines such as LIF and IL-1β.

Using a CCK8 proliferation assay and a trans-well invasion assay, we found that hCG-treated PBMC have no effect on JAR cell proliferation, whereas hCG-activated PBMC dramatically enhanced dramatically the invasive activity of JAR cells compared to control or hCG only, indicating that hCG can induce PBMC to become trophoblast cell invasion activators. Trophoblast cells are highly invasive due to the secretion of extracellular proteases like MMP, mediating extracellular matrix degradation, and balancing TIMP [24–26]. Successful implantation and trophoblast invasion are closely linked to the expression of MMP, which can degrade basement membranes. The two gelatinases MMP-2 and MMP-9, which digest type IV collagen, the main component of the basement membrane, are expressed by trophoblast cells and considered key enzymes in trophoblast invasion [27–29]. TIMP-1 and TIMP-2 are considered responsible for protecting vessels and maintaining blood vessel integrity as MMP inhibitors [30]. In particular, the role of TIMP-1 might be more distinct since it has also been shown to inhibit other metalloproteinases [31–33]. Hence, the local balance between MMP and TIMP at the invasive site is a highly regulated process mediated by the spatiotemporal expression of the MMP involved in ECM remodeling and requires the appropriate trophoblast–endometrium interaction [34].

To elucidate the potential effector molecules involved in PBMC activated by hCG-induced cell invasion, the expressions of MMP-2 and MMP-9 as well as their inhibitors TIMP-1 and TIMP-2 were investigated using western blotting and real-time PCR. Our study results showed that MMP-2 and MMP-9 expressions in JAR cells were significantly increased and TIMP-1 and TIMP-2 expressions were decreased with the supernatant fluid in a dose-dependent manner, which was consistent with our results from an invasion assay of JAR cells. Furthermore, studies have shown that VEGF plays an important role in trophoblast invasion, maternal vascular transformation, and fetoplacental vascular differentiation, and development and was able to enhance MMP-2 and MMP-9 activity and trophoblast invasion ability [35–37]. On the other hand, the VEGF–ligand receptor system reportedly influences cell differentiation and permeability of the uterine epithelium and the fetal membranes (trophoblast), thereby affecting placental development and growth [38]. Crocker et al. [39] found that VEGF promoted the *in vitro* syncytialization of first-trimester cytotrophoblasts. Therefore, VEGF and its receptor not only stimulate angiogenesis but also regulate trophoblast differentiation and invasion. All of these findings suggest that PBMC activated by hCG could promote trophoblast cell invasion, an effect that might partially occur through the upregulation of MMP-2, MMP-9, and VEGF expression and downregulation of TIMP-1 and TIMP-2 expression.

Recent focus has been placed on patients with recurrent implantation failure who fail to become pregnant despite repeated transfers of high-quality embryos. Although ovarian sex steroid hormones are mainly used to ameliorate endometrial receptivity, there is not yet any effective therapy for those patients who do not respond to hormonal control. Yoshioka et al. [40] developed a new immunotherapy for patients with repeated implantation failure. The intrauterine administration of autologous PBMC, which was activated by incubation with hCG for 48 h, effectively improved the implantation, clinical pregnancy, and live birth rates of patients with repeated in vitro fertilization failure. Later, Okitsu et al. [41] found that intrauterine administration of freshly isolated PBMC effectively improved embryo implantation in patients receiving frozen/thawed embryo transfer after three or more repeated in vitro fertilization failures. We previously proposed that the intrauterine administration of hCG-activated murine PBMC could promote embryonic implantation by regulating endometrial receptivity, providing a possible mechanism of this new immunotherapy [12]. However, the effects of hCG on PBMC function derived from non-pregnant women *in vitro* and the physiological effect of activated PBMC on trophoblasts at the early implantation site remain unclear. In this regard, this study provides important evidence that hCG is a potent candidate for enhancing the promoting effect of patients' PBMC *in vitro* and that these activated PBMC around the embryo could promote embryonic invasion for clinical application.

## Conclusion

In conclusion, this study demonstrated that, as a major embryonal signal, hCG could activate PBMC to induce the secretion of several cytokines that promote embryonic invasion. These findings suggest a positive feedback loop in which PBMC at the implantation site is activated by hCG, which is secreted from the embryo, and then these activated PBMC facilitate embryonic invasion. In addition, results from this study suggest that patients’ PBMC activated by hCG might be used for therapy in patients suffering from implantation failure.
